# Inflammation and Vitamin C in Women with Prenatal Depression and Anxiety: Effect of Multinutrient Supplementation

**DOI:** 10.3390/antiox12040941

**Published:** 2023-04-17

**Authors:** Anitra C. Carr, Hayley A. Bradley, Emma Vlasiuk, Hayley Pierard, Jessica Beddow, Julia J. Rucklidge

**Affiliations:** 1 Nutrition in Medicine Research Group, Department of Pathology and Biomedical Science, University of Otago, Christchurch 8011, New Zealand; emma.vlasiuk@otago.ac.nz; 2 School of Psychology, Speech and Hearing, University of Canterbury, Christchurch 8041, New Zealand; hayley.bradley@canterbury.ac.nz (H.A.B.); julia.rucklidge@canterbury.ac.nz (J.J.R.); 3 Centre for Postgraduate Nursing Studies, University of Otago, Christchurch 8011, New Zealand

**Keywords:** inflammatory, perinatal, mood disorders, micronutrients, interleukin-6, C-reactive protein, tumour necrosis factor, ascorbic acid, body weight

## Abstract

Elevated inflammation has been associated with adverse mood states, such as depression and anxiety, and antioxidant nutrients, such as vitamin C, have been associated with decreased inflammation and improved mood. In the current study comprising a cohort of pregnant women with depression and anxiety, we hypothesised that elevated inflammation would be associated with adverse mood states and inversely associated with vitamin C status and that multinutrient supplementation would optimise vitamin concentrations and attenuate inflammation. Sixty-one participants from the NUTRIMUM trial had blood samples collected between 12 and 24 weeks gestation (baseline) and following 12 weeks of daily supplementation with a multinutrient formula containing 600 mg of vitamin C or active placebo. The samples were analysed for inflammatory biomarkers (C-reactive protein (CRP) and cytokines) and vitamin C content and were related to scales of depression and anxiety. Positive correlations were observed between interleukin-6 (IL-6) and all of the mood scales administered (*p* < 0.05), including the Edinburgh Postnatal Depression Scale, the Clinical Global Impressions—Severity Scale, the Montgomery and Åsberg Depression Rating Scale, the Depression Anxiety Stress Scale 21, and the Generalized Anxiety Disorder-7 (GAD-7). CRP correlated weakly with GAD-7 (*p* = 0.05). There was an inverse correlation between CRP and the vitamin C status of the cohort (*p* = 0.045), although there was no association of the latter with the mood scales (*p* > 0.05). Supplementation with the multinutrient formula resulted in a significant increase in the vitamin C status of the cohort (*p* = 0.007) but did not affect the inflammatory biomarker concentrations (*p* > 0.05). In conclusion, greater systemic inflammation was associated with worse mood states; however, 12-week multinutrient supplementation did not alter inflammatory biomarker concentrations. Nevertheless, the vitamin C status of the cohort was improved with supplementation, which may aid pregnancy and infant outcomes.

## 1. Introduction

Prenatal depression is characterised by the onset of a major depressive episode during pregnancy and is a leading cause of maternal morbidity and mortality [1]. Prenatal depression is often associated with severe anxiety, the prevalence of which can be higher than that of depression itself [2,3]. Left untreated, depression and anxiety during the prenatal period can have severe and long-term consequences not only for the women affected but also for their infant and the rest of their family [4]. Many biological mechanisms have been proposed to explain the aetiology of mood disorders, one of which is elevated inflammation. A recent meta-analysis of 107 studies indicated elevated systemic inflammatory biomarkers in people with depression relative to healthy controls [5]. The rationale that inflammation may play a role in the development and maintenance of prenatal depression is therefore plausible and is supported by research indicating that systemic inflammation may be associated with both prenatal depression and adverse pregnancy outcomes, such as pre-eclampsia, preterm birth and gestational diabetes [6,7,8]. Thus, a better understanding of the psychoimmunology of pregnancy is necessary to help reduce the burden of prenatal mental illness and increase the likelihood of successful pregnancy outcomes [9].

It is well known that nutritional demands increase during pregnancy and lactation due to enhanced nutrient requirements for foetal/infant growth and development, demands that may result in multiple nutritional deficiencies for the mother. Recent evidence has identified an association between poor diet quality, nutritional deficiencies, and prenatal depression [10,11]. Vitamin C, in particular, is an essential nutrient found in high concentrations in the brain and associated glands [12]. This vitamin is a potent antioxidant and has vital roles in catecholamine and neuropeptide hormone synthesis, as well as other important functions within the central nervous system [13,14]. Circulating vitamin C concentrations decrease during pregnancy [15,16], which could lead to a deficiency if baseline levels are low. Vitamin C deficiency has been associated with low mood and depression [17,18], and intervention studies have indicated improvements in mood following vitamin C administration [19], particularly in people not currently taking anti-depressants [20].

There is some evidence that vitamin C may have anti-inflammatory properties, with studies indicating that vitamin C administration reduces biomarkers of inflammation, such as the acute phase reactant C-reactive protein (CRP) and pro-inflammatory cytokines, e.g., interleukin-6 (IL-6), in people with elevated inflammation [21,22]. The mechanisms involved are uncertain but may relate to vitamin C’s antioxidant properties, as there is a strong link between inflammation and oxidative stress in many disorders, including depression [23]. Therefore, in the current study, we aimed to investigate associations between inflammatory biomarkers (specifically CRP, IL-6, IL-10 and tumour necrosis factor alpha (TNF-α)), vitamin C status, and mood disorders in a cohort of women with prenatal depression and anxiety before and after receiving a multinutrient supplement or active placebo for 12 weeks [24]. We hypothesized that elevated inflammation would be associated with adverse mood states and inversely associated with vitamin C status and that multinutrient supplementation would optimize vitamin concentrations and attenuate inflammation. The multinutrient formula chosen for the study was a broad-spectrum supplement that has previously been shown to improve psychological illnesses in various cohorts [25].

## 2. Materials and Methods

### 2.1. Study Participants

This study comprised a nested prospective analysis of the vitamin C status and inflammatory biomarker concentrations of a cohort of pregnant women recruited as part of the NUTRIMUM trial, an RCT designed to assess the effect of a broad-spectrum multinutrient supplement on the symptoms of antenatal depression and anxiety. Detailed information about the study design can be found in the published study protocol [24]. The trial was approved by the New Zealand Southern Health and Disability Ethics Committee (16/STH/187) and was registered with the Australian and New Zealand Clinical Trial Registry (ACTRN12617000354381). Pregnant women between 12 and 24 weeks gestation were recruited. The inclusion criteria were: aged ≥16 years; low-risk singleton pregnancy; free from psychiatric medication for 4 weeks; a score of ≥13 on the Edinburgh Postnatal Depression Scale during their second trimester; and deemed reliable and compliant with the protocol. The exclusion criteria included regular vomiting; current/recent significant pregnancy complications; known foetal abnormalities; serious current or historical medical condition; known allergy to the ingredients of the intervention; known metabolic condition; untreated or unstable thyroid disease; known neurological disorder; and a desire to continue taking prenatal supplements that either exceed the upper limit or were not required for medical purposes (decisions were discussed and made on a case-by-case basis).

**Figure 1:**
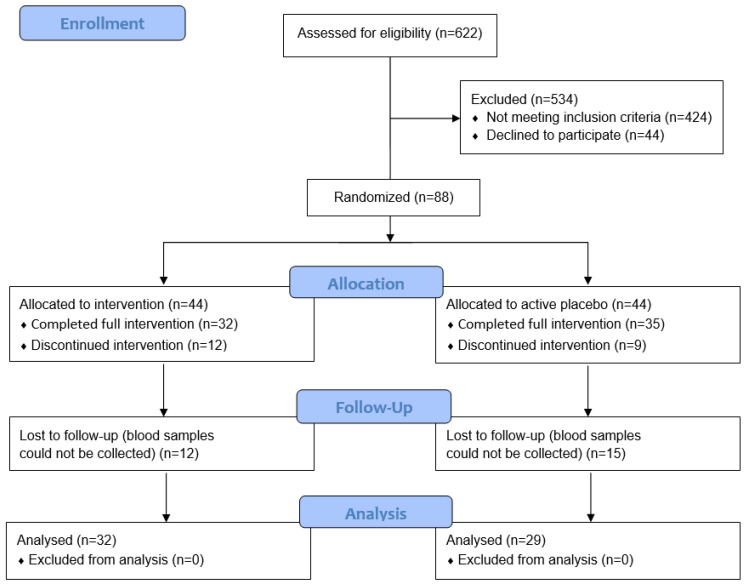
CONSORT flow diagram.

Power calculations for the main trial were based on the Edinburgh Postnatal Depression Scale (EPDS) as the primary outcome measure [24]. These indicated that a total of 90 participants (45 per group) would be required to detect a medium effect size of Cohens *d* = 0.6 at 80% power and a significance level of 0.05. The final trial comprised 88 pregnant women; however, for the nested biomarker study we were able to collect blood samples from only 61 of the participants due to limitations around nationwide COVID-19-related lockdowns and social restrictions, and some participants also declined to provide blood samples (Figure 1).

### 2.2. Intervention

**Figure 2:**
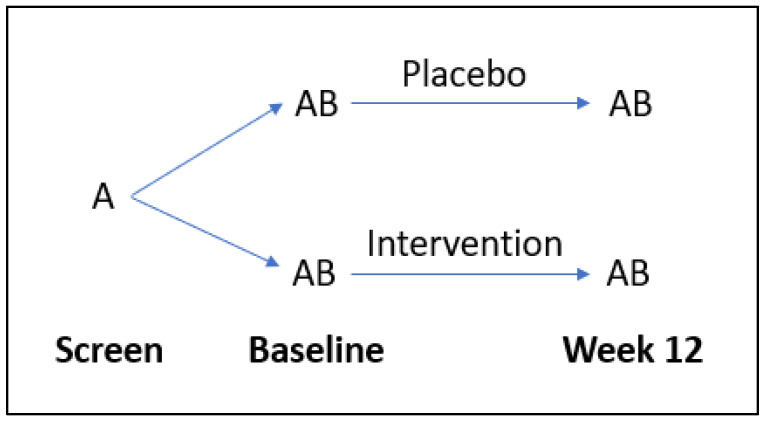
Flow diagram of study design. A = questionnaires, B = laboratory tests.

Participants were randomly allocated (1:1) to receive either a broad-spectrum multinutrient formula (Daily Essential Nutrients supplied by Hardy Nutritionals) or an active control formula containing iodine and riboflavin for a 12-week period (Figure 2). The full daily dose of 12 capsules was taken as four capsules three times daily. The complete list of intervention ingredients is available from the published study protocol [24]. Each capsule contained 50 mg of vitamin C (as ascorbic acid); thus, the full dose of 12 capsules comprised 600 mg of vitamin C daily.

### 2.3. Data Collection

Demographic characteristics, including age, ethnicity, household income, and marital status, were collected in a screening assessment. Gestation, body weight and blood pressure were also measured at the baseline and week 12 clinic visits. Dietary quality was monitored at baseline and week 12 using the Dietary Screening Tool, a 24-item self-report questionnaire that assesses dietary intake and identifies those at nutritional risk [26]. The Dietary Screening Tool has a total score of 100, with higher scores indicating a healthier dietary pattern; <60 indicates nutritional risk, 60–75 indicates potential nutritional risk, and >75 indicates no nutritional risk. Some participants continued or started the uptake of single nutrients (e.g., iron) if medically indicated. Nine participants (15%) reported taking vitamin-C-containing supplements at baseline or earlier, and six (10%) reported this at other times during the study, mostly sporadically.

### 2.4. Mood Questionnaires

The Edinburgh Postnatal Depression Scale (EPDS) is a 10-item self-report questionnaire designed to assess the affective and cognitive symptoms of depression and anxiety over the previous 7 days [27]. Each item on the EPDS is rated on a 4-point scale, with a total score ranging from 0 to 30, with greater levels of distress indicated by higher scores. A cut-off score of ≥13 was used in this study to identify the incidence of perinatal depression.

The Clinical Global Impressions—Severity Scale (CGI-S) is a clinician-rated evaluation of symptom severity ranging from 1 (normal, not ill) to 7 (very severely ill) [28]. The CGI-S was used to assess the severity of mood, anxiety, and global functioning.

The Montgomery and Åsberg Depression Rating Scale (MADRS) is a 10-item clinician-rated scale that assesses the severity, frequency and duration of depressive symptoms over the previous week [29]. Each item is rated on a 7-point scale ranging from 0 to 5, with a maximum score of 60. A greater score indicates more severe symptoms.

The Depression Anxiety Stress Scale 21 (DASS-21) is a 21-item self-report questionnaire with three 7-item subscales measuring the severity of depression, anxiety, and stress, each summing to a maximum score of 21 with more severe symptoms identified with higher scores [30]. Each item on the DASS-21 is rated on a 4-point scale ranging from 0 (did not apply to me at all) to 3 (applied to me very much, or most of the time). The DASS-21 provides clinical cut-off scores for each subscale ranging from normal to extremely severe [31].

The Generalized Anxiety Disorder-7 (GAD-7) is a 7-item self-report measure of generalised anxiety symptoms over the past 2 weeks. Each item is rated on a 4-point scale ranging from 0 (not at all) to 3 (nearly every day) [32]. Total scores range from 0 to 21, with more severe symptoms identified with higher scores. The GAD-7 comprises the following cut-off scores: normal: 0–4; mild: 5–9; moderate: 10–14 and severe: 15–21.

### 2.5. Sample Collection and Biomarker Analyses

Fasting blood samples were collected at baseline and post-intervention for the assessment of inflammatory biomarkers, including C-reactive protein (CRP), interleukin-6 (IL-6), interleukin-10 (IL-10), and tumour necrosis factor-α (TNF-α). For CRP, clinically relevant thresholds range from <1.0 mg/L (low) to >3 mg/L (high) or >10 mg/L (very high), with values > 50 mg/L generally indicating infection. High-sensitivity CRP was measured using end-point nephelometry; one baseline blood sample was not able to be obtained (*n* = 60). IL-6, IL-10, and TNF-α were measured in a subgroup of participants (*n* = 41) using enzyme immunoassays (BioLegend, San Diego, CA, USA). These assays were carried out by Canterbury Health Laboratories, an International Accreditation New Zealand (IANZ) laboratory.

Fasting blood samples for vitamin C analysis were collected into heparin tubes and rapidly processed for stable storage at −80 °C [33]. These samples were analysed in bulk at the end of the study intervention period using high-performance liquid chromatography (HPLC) as described previously [34]; one baseline blood sample was not able to be obtained (*n* = 60). The reference range for vitamin C is 26–85 µmol/L, with relevant thresholds being ≤11 µmol/L (deficiency), ≤23 µmol/L (hypovitaminosis C), ≥50 µmol/L (adequate), and ≥70 µmol/L (saturating).

### 2.6. Statistical Analyses

Data are presented as the median and interquartile range (Q1, Q3) for continuous variables and as *n* (%) for categorical variables. Linear regressions were carried out using Pearson’s *r*, and non-parametric correlations were carried out using Spearman’s *r*, with *p* < 0.05 considered statistically significant. Group comparisons were carried out using non-parametric Mann–Whitney U tests or Wilcoxon matched-pairs signed-rank tests. All statistical analyses and graphical representations were carried out using GraphPad Prism version 9.1.2 (GraphPad, San Diego, CA, USA).

## 3. Results

### 3.1. Participant Characteristics

The cohort comprised 61 women of a median age of 32 (range 20–40) years, predominantly NZ European ethnicity (79%), married or in a de facto relationship (88%), and living in a mid-high income household (82%; Table 1). At baseline, the median gestation was 17 (15, 22) weeks, and the participants weighed a median of 73 (62, 88) kg. By week 12 (30 (27, 33) weeks gestation), the participants weighed a median of 78 (68, 90) kg, a gain of 6.6 (5.4, 8.9) kg over the 12-week study period. There was no change in diet quality over the 12-week study period (*p* = 0.24). The various mood scales showed mild to moderate disorder in the cohort at baseline, with scores as follows: EPDS, 15 (14, 18); MADRS, 18 (14, 23); DASS-21, 19 (14, 26); GAD-7, 9 (7, 13); and CGI-S, 4 (3, 4).

### 3.2. Relationship of Inflammation with Mood

**Figure 3:**
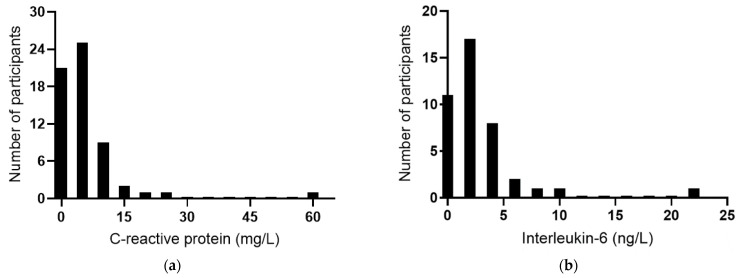

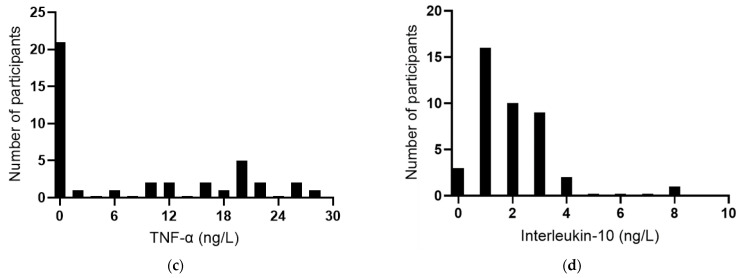
Baseline inflammatory biomarker status in the study cohort. Frequency distributions of: (**a**) C-reactive protein (*n* = 60), (**b**) interleukin-6 (*n* = 41), (**c**) tumour necrosis factor-alpha (TNF-α; *n* = 40), and (**d**) interleukin-10 (*n* = 41).

The baseline plasma concentrations of the acute-phase reactant CRP were a median of 4.3 (2.0, 7.1) mg/L (*n* = 60). Among the participants, 58% had CRP concentrations >3 mg/L, 12% had concentrations ≥10 mg/L, and there was a clear outlier of 59 mg/L (Figure 3a), indicating possible infection or an inflammatory condition in this participant. IL-6 concentrations were a median of 2.2 (0.7, 3.5) ng/L (*n* = 51); once again, there was a clear outlier of 22 ng/L (Figure 3b). TNF-α concentrations were a median of 0.8 (0, 19) ng/L (Figure 3c), and IL-10 concentrations were a median of 1.5 (0.9, 2.5) ng/L (Figure 3d). There was a positive correlation between CRP and IL-6 concentrations (*r* = 0.739, *p* < 0.0001), although the removal of the common outlier resulted in the loss of significance (*r* = 0.109, *p* = 0.51). There were no significant correlations between CRP and TNF-α or IL-10. There was a positive correlation between TNF-α and IL-10 (*r* = 0.571 *p* = 0.0001) and inverse correlations between IL-6 and TNF-α (*r* = −0.514, *p* = 0.0007) and between IL-6 and IL-10 (*r* = −0.316 *p* = 0.044).

**Figure 4:**
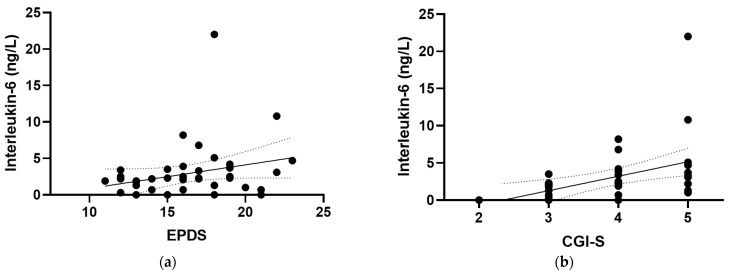

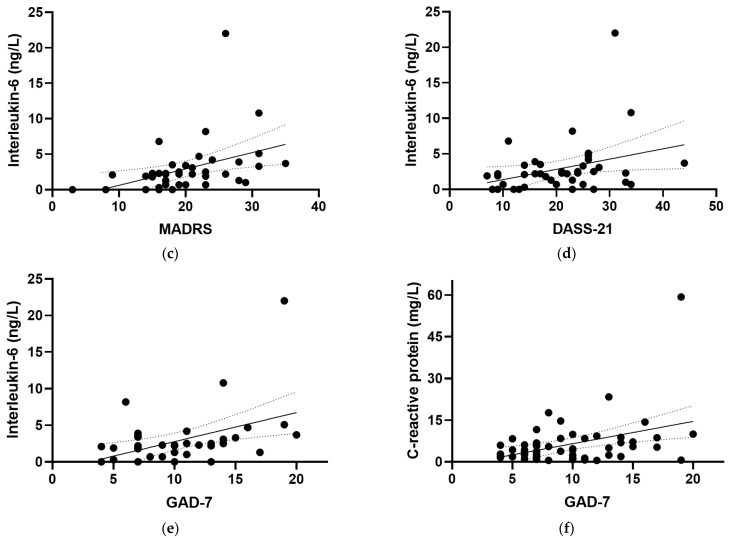
Correlations of inflammatory biomarkers with mood scores. Correlations of interleukin-6 with (**a**) the Edinburgh Postnatal Depression Scale (EPDS; *r* = 0.319, *p* = 0.04), (**b**) the Clinical Global Impressions—Severity Scale (CGI-S; *r* = 0.528, *p* < 0.001), (**c**) the Montgomery and Åsberg Depression Rating Scale (MADRS; *r* = 0.563, *p* < 0.001), (**d**) the Depression Anxiety Stress Scale 21 (DASS-21; *r* = 0.379, *p* = 0.015), and (**e**) the Generalized Anxiety Disorder-7 (GAD-7; *r* = 0.442, *p* = 0.005). (**f**) Correlation of C-reactive protein with GAD-7 (*r* = 0.260, *p* = 0.05). Dashed lines indicate 95% confidence intervals.

At baseline, positive correlations were observed between IL-6 and all of the mood scores assessed in the study (Figure 4), including the EPDS (*r* = 0.319, *p* = 0.04), the CGI-S (*r* = 0.528, *p* <0.001), the MADRS (*r* = 0.563, *p* < 0.001), the DASS-21 (*r* = 0.379, *p* = 0.015), and the (GAD-7; *r* = 0.442, *p* = 0.005). There were also positive correlations observed between IL-6 and the mood and anxiety subscales of the CGI-S (*r* = 0.396, *p* = 0.01 and *r* = 0.371, *p* = 0.017, respectively) and the depression and anxiety subscales of the DASS-21 (*r* = 0.313, *p* = 0.047 and *r* = 0.389, *p* = 0.012, respectively). Removal of the outlying IL-6 value retained significance for the CGI-S, MADRS, DASS-21 and GAD-7 correlations, with a small loss of significance for the EPDS (*p* = 0.057). CRP correlated weakly with GAD-7 (*r* = 0.260, *p* = 0.05; Figure 4f), a correlation which was lost following the removal of the outlying CRP value (*p* = 0.1). Plasma TNF-α and IL-10 concentrations did not correlate with any of the mood scores at baseline (*p* > 0.05).

### 3.3. Relationship of Inflammation with Vitamin C Status

**Figure 5:**
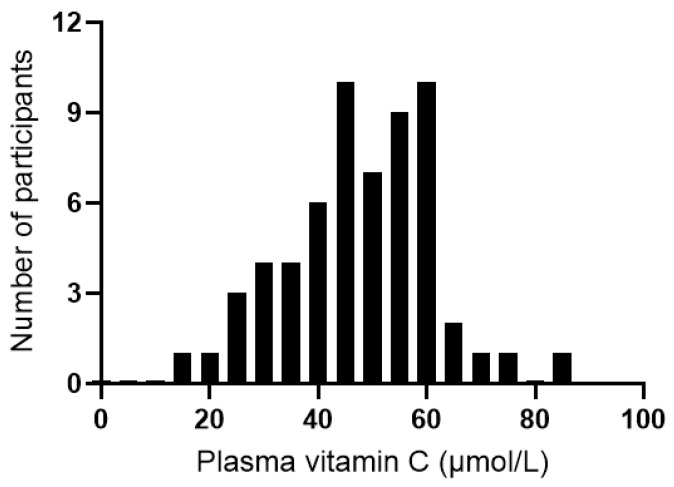
Distribution of plasma vitamin C concentrations of the study cohort at baseline (*n* = 60).

The median plasma vitamin C status of the cohort at baseline was 48 (40, 57) µmol/L (range 16–84 µmol/L; Figure 5). Of the 60 participants assessed at baseline, 31 (52%) had inadequate vitamin C status (<50 µmol/L), and 2 (3%) had hypovitaminosis C (≤23 µmol/L). There was an inverse correlation between vitamin C status and CRP concentrations (*r* = −0.259, *p* = 0.045); however, removal of the CRP outlier resulted in a loss of significance (*r* = −0.230, *p* = 0.079). There were no correlations between vitamin C status and IL-6, TNF-α, or IL-10 concentrations (*p* > 0.05). Plasma vitamin C concentrations did not correlate with any of the mood scores at baseline (*p* > 0.05).

**Figure 6:**
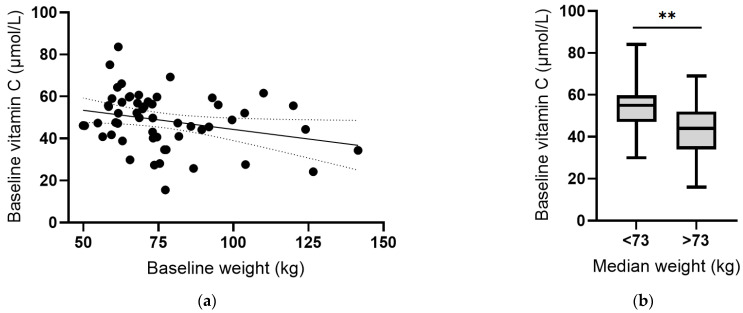

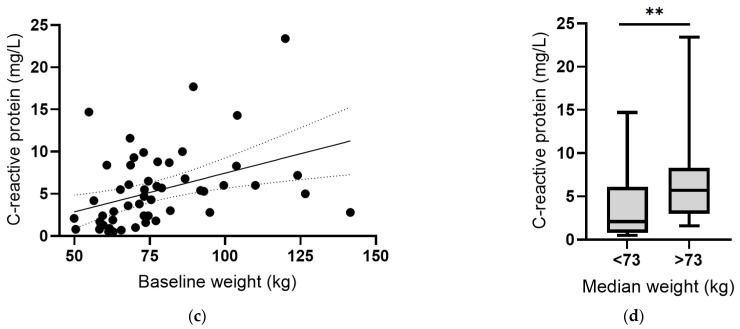
Vitamin C and C-reactive protein concentrations relative to body weight. (**a**) Inverse correlation between vitamin C status and weight (*r* = −0.287, *p* = 0.03). (**b**) Vitamin C status relative to median body weight (** *p* = 0.001). (**c**) Positive correlation between CRP and weight (*r* = 0.501, *p* = 0.0001). (**d**) CRP concentration relative to median body weight (** *p* = 0.004). Dashed lines indicate 95% confidence intervals. Box plots represent median with 25th and 75th percentiles as boundaries, and whiskers indicate maximum and minimum values.

Instead, there was an inverse correlation between plasma vitamin C concentrations and body weight at baseline (*r* = −0.287, *p* = 0.03; Figure 6a). Assessing vitamin C status relative to median body weight indicated a significant difference (*p* = 0.001; Figure 6b). Conversely, there was a positive correlation between CRP concentrations and body weight, which was strengthened following removal of the outlier (*r* = 0.501, *p* = 0.0001; Figure 6c), and a significant difference between CRP concentrations above and below the median body weight (*p* = 0.004; Figure 6d). Thus, associations between vitamin C and CRP may be related to body weight. The plasma concentrations of the other cytokines (IL-6, IL-10 and TNF-α) did not correlate with baseline body weight (*p* > 0.05).

### 3.4. Effect of Multinutrient Supplementation on Vitamin C Status and Inflammation

**Figure 7:**
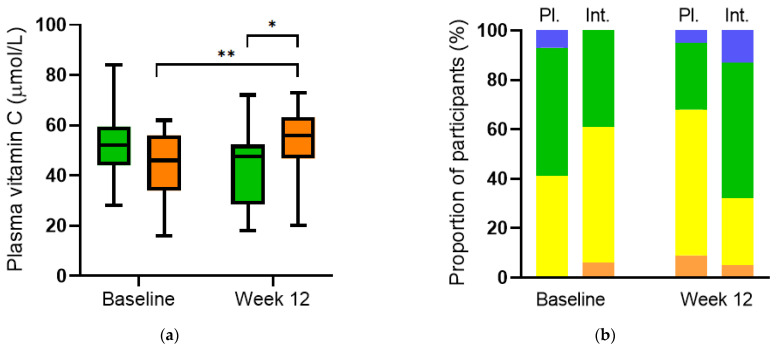
Plasma vitamin C concentrations before and after intervention. (**a**) Vitamin C increased over time in the intervention group (orange boxes; ** *p* = 0.007) and relative to placebo at week 12 (green boxes; * *p* = 0.01). (**b**) Categories of vitamin C status: hypovitaminosis C (≤23 µmol/L; orange bars), inadequate (<50 µmol/L; yellow bars), adequate (≥50 µmol/L; green bars), saturating (≥70 µmol/L; blue bars). Pl., placebo; Int., intervention.

Supplementation of participants (*n* = 32) with multinutrient intervention comprising a total of 600 mg/day of vitamin C for 12 weeks resulted in a significantly higher vitamin C status relative to baseline (56 (47, 63) µmol/L vs 46 (34, 56) µmol/L, respectively; *p* = 0.007). Furthermore, a significantly higher vitamin C status was observed in the intervention group relative to the placebo group (*n* = 29) at week 12 (56 (47, 63) µmol/L vs. 48 (29, 52) µmol/L, respectively; *p* = 0.01; Figure 7a). Categorisation of the vitamin C concentrations in the different groups showed an increase in the proportion of participants with adequate and saturating vitamin C status (≥50 µmol/L) in the intervention group over 12 weeks (from 39% to 68%) and an increase in the proportion of participants with inadequate vitamin C status (<50 µmol/L) in the placebo group (from 41% to 68%; Figure 7b). 

The inflammatory biomarker concentrations did not vary significantly in the two groups over the 12-week study period (*p* > 0.05), and the multinutrient intervention had no effect on the inflammatory biomarkers relative to the placebo group at week 12 (*p* > 0.05; Table 2). This may have been due to the majority of participants not having dramatically elevated inflammation at baseline. In support of this notion, the participant with elevated inflammation at baseline (59 mg/L CRP and 22 ng/L IL-6) exhibited a clear decrease in these markers following supplementation with the multinutrient formula (to 32 mg/L CRP and 7.5 ng/L IL-6), as well as an increase in vitamin C status from an inadequate 30 µmol/L to 57 µmol/L. This nested study was not powered to detect differences in mood scores between the two subgroups following supplementation. Of note, though, there was a trend towards decreases in anxiety as assessed by the CGI-S anxiety subscale with increases in vitamin C status (*r* = −0.273, *p* = 0.076) over the 12-week study period.

## 4. Discussion

Our study comprising a cohort of women with prenatal depression and anxiety showed higher concentrations of inflammatory biomarkers, predominantly IL-6, in those with more elevated depression and anxiety, assessed using a variety of mood scales. Although TNF-α is also a pro-inflammatory cytokine, there was an inverse association between IL-6 and TNF-α, as IL-6 can downregulate TNF-α [35], and no association of TNF-α with any of the mood scales. In contrast, there was a positive correlation between IL-6 and the acute-phase reactant CRP, as IL-6 can upregulate the acute phase response [35], although this association was primarily driven by an outlying participant with significantly elevated inflammation, possibly due to an infection or underlying inflammatory condition. In the cohort as a whole, higher CRP concentrations correlated with elevated anxiety as assessed by the GAD-7 scale. Inflammation and the accompanying elevation of inflammatory cytokines have been associated with not only mood disorders, including perinatal depression, but also with foetal and maternal morbidities such as pre-eclampsia, preterm birth, and gestational diabetes [8].

Vitamin C deficiency is associated with low mood and depression [17,18]; however, no associations were observed in our cohort between the vitamin C status of the participants and the various mood scores at baseline. This is likely due to the low prevalence of hypovitaminosis C (3%) and the complete lack of deficiency in the mostly middle-high income cohort. Thus, further research investigating the associations between vitamin C and prenatal depression and anxiety should ideally be carried out in cohorts at risk of vitamin C deficiency at baseline, such as those from low socioeconomic populations or those with higher baseline body weight [36,37]. Vitamin C concentrations are known to decrease with increasing body weight [38]. Likewise, longer gestational time, and the associated increase in weight, are associated with the declining vitamin C status of the mother [15,16]. In our study, we observed an inverse correlation between vitamin C status and body weight early during pregnancy. It is, therefore, likely that the decrease in vitamin C status observed in the placebo group after 12 weeks was contributed to by the increase in body weight over the study period. As such, women with higher body weight early in pregnancy may be at enhanced risk of developing vitamin C insufficiency during their pregnancy. Furthermore, deficient vitamin C status in pregnant women can predispose their infants to significant hypovitaminosis C at birth, which, in turn, can increase the infants’ risk of adverse outcomes [39].

Supplementation of the participants with the vitamin C-containing multinutrient formula increased the vitamin C status of the participants (from 46 to 56 µmol/L), despite their increase in weight (from 73 to 78 kg) over the 12-week intervention period. Nevertheless, approximately one-third of the participants still had inadequate vitamin C status (i.e., <50 µmol/L), despite consuming a relatively large daily dose of 600 mg of the vitamin. This could be due to a number of factors, such as vomiting, non-compliance, or low bioavailability of the supplement. Vitamin C has important roles to play in epigenetic regulation during development [40] and is also a cofactor for collagen synthesis, with supplementation showing positive benefits regarding attenuating premature rupture of membranes (RR 0.66, 95% CI 0.48–0.91) [41]. Low vitamin C status has also been associated with higher odds of developing pre-eclampsia (OR 3.8, 95% CI 1.7–8.8) [42]. The epigenetic and other related cofactor functions of vitamin C are dependent on adequate concentrations of the vitamin [43]. As such, lowered vitamin C status associated with higher body weight during pregnancy may compromise the healthy development of the foetus and potentially result in long-term adverse health outcomes. Thus, ensuring adequate vitamin C status, based on body weight, throughout pregnancy may help improve both birth and infant outcomes.

Multivitamin supplementation of HIV-positive pregnant women has been shown to improve mood and quality of life [44]. HIV infection is known to both increase inflammation (as assessed by CRP concentrations) and deplete antioxidant nutrients such as vitamin C [45]. In our study, the multinutrient intervention did not appear to have any overall effect on the inflammatory biomarker concentrations, likely due to these not being hugely elevated at baseline in a majority of the participants. One notable exception was the participant with elevated inflammation (CRP and IL-6) at baseline who exhibited a clear decrease in these markers following supplementation with the multinutrient formula, as well as an increase in vitamin C status from an inadequate concentration of 30 µmol/L to an adequate concentration of 57 µmol/L. Thus, future research would benefit from a focus on participants with both depleted micronutrient status and elevated inflammatory biomarkers at baseline.

Improvements in mood were observed in the full cohort over the 12-week study period; these have been reported elsewhere [46]. There was also a modest inverse association between changes in vitamin C status and changes in anxiety, as assessed by the CGI-S anxiety subscale, over the 12-week study period. Vitamin C has close associations with the stress hormone adrenaline, being a cofactor in the stress response [47,48,49,50]. Thus, it is conceivable that the stress response can deplete vitamin C levels, requiring supplementation and/or an improvement in mood to restore adequate vitamin C status. This may be particularly problematic in women who already have low vitamin C status, resulting in a potential deficiency of the vitamin if stressed for a prolonged period of time.

There are a number of limitations of our study, a major factor being the lower-than-anticipated participant numbers, primarily due to nationwide COVID-related restrictions. As the vitamin C and inflammatory biomarker data were secondary outcome measures, the participants were not specifically screened for low vitamin C status or elevated inflammation, thus reducing the power to detect potential effects of the multinutrient intervention. Furthermore, there are many confounding factors that can influence both vitamin C and inflammatory biomarker concentrations [37,51], as well as mood states, which have not been assessed as part of this study. A number of factors that are known to be predictors of treatment response are being included in the analyses of the main trial findings due to their potential influence on mood outcomes. The impact of the multinutrient supplement on the status of other micronutrients (e.g., vitamins B12 and D, iron, copper, and zinc) will also be reported in the main trial publication.

## 5. Conclusions

In our cohort of women with elevated depression and anxiety, specific pro-inflammatory cytokines, i.e., IL-6 and CRP, were positively associated with more elevated scores of depression and/or anxiety. Although the overall vitamin C status of the cohort was adequate, higher body weight was associated with lower vitamin C status, which can potentially put the mother and foetus at increased risk of deficiency as the pregnancy progresses. Supplementation with a multinutrient formula increased the vitamin C status of the cohort, despite pregnancy-related weight gain over the 12-week intervention period, which may aid both pregnancy and infant outcomes. The intervention did not, however, affect systemic inflammatory biomarker concentrations, possibly due to a lack of sufficient elevation of the biomarkers at baseline. Thus, future research should focus on cohorts that have both depleted micronutrient status and elevated inflammation at baseline.

## Figures and Tables

**Table 1:** Participant characteristics at baseline and week 12 (*n* = 61).

Characteristics	Baseline	Week 12
Age, years	32 (27, 35)	
Ethnicity:		
NZ European	48 (79)	
Māori/Pasifika	6 (10)	
Asian/Other	7 (11)	
Household income:		
Low (0–40 K)	11 (18)	
Mid (40–80 K)	20 (33)	
High (80+ K)	30 (49)	
Relationship status:		
Single	7 (11)	
Married	32 (52)	
Defacto	22 (36)	
EPDS	15 (14, 18)	
Gestation, weeks	17 (15, 22)	30 (27, 33)
Weight, kg	73 (62, 88)	78 (68, 90)
Systolic BP, mmHg	117 (105, 124)	116 (108, 126)
Diastolic BP, mmHg	77 (72, 83)	84 (79, 88)
Diet quality ^1^	65 (59, 71)	68 (60, 71)

Data represent median (Q1, Q3) or *n* (%). ^1^ Determined using the Dietary Screening Tool (maximum score is 100). EPDS, Edinburgh Postnatal Depression Scale (≥13 indicates depression).

**Table 2:** Inflammatory biomarker concentrations at baseline and week 12.

	Placebo (*n* = 21)		Intervention (*n* = 20)	
Inflammatory Markers	Baseline	Week 12	Baseline	Week 12
CRP (mg/L)	4.2 (1.8, 6.1)	2.6 (1.2, 5.9)	5.0 (2.4, 8.7)	5.1 (1.3, 7.1)
IL-6 (ng/L)	2.3 (0.7, 4.1)	3.5 (0.5, 5.0)	2.2. (0.8, 2.5)	3.5 (0.8, 5.2)
TNF-α (ng/L)	0 (0, 19)	0.4 (0, 11)	1.6 (0, 18)	2.1 (0, 18)
IL-10 (ng/L)	1.5 (0.8, 2.9)	2.2 (1.2, 2.8)	1.6 (1.0, 2.5)	1.6 (1.0, 3.2)

Data represent median (Q1, Q3). CRP, C-reactive protein; IL-6, interleukin 6; TNF-α, tumour necrosis factor-α; IL-10, interleukin 10. There were no statistically significant differences between baseline and week 12 for either group (*p* > 0.05) or between intervention and placebo at week 12 (*p* > 0.05).

## Data Availability

Data are available upon reasonable request for inclusion in meta-analyses.

## References

[1] Mathers C.D., Loncar D. (2006). Projections of global mortality and burden of disease from 2002 to 2030. PLoS Med..

[2] American Psychiatric Association (2013). Diagnostic and Statistical Manual of Mental Disorders.

[3] Signal T.L., Paine S.J., Sweeney B., Muller D., Priston M., Lee K., Gander P., Huthwaite M. (2017). The prevalence of symptoms of depression and anxiety, and the level of life stress and worry in New Zealand Māori and non-Māori women in late pregnancy. Aust. N. Z. J. Psychiatry.

[4] Letourneau N.L., Dennis C.L., Cosic N., Linder J. (2017). The effect of perinatal depression treatment for mothers on parenting and child development: A systematic review. Depress. Anxiety.

[5] Osimo E.F., Pillinger T., Rodriguez I.M., Khandaker G.M., Pariante C.M., Howes O.D. (2020). Inflammatory markers in depression: A meta-analysis of mean differences and variability in 5,166 patients and 5,083 controls. Brain Behav. Immun..

[6] Cassidy-Bushrow A.E., Peters R.M., Johnson D.A., Templin T.N. (2012). Association of depressive symptoms with inflammatory biomarkers among pregnant African-American women. J. Reprod. Immunol..

[7] Christian L.M., Franco A., Glaser R., Iams J.D. (2009). Depressive symptoms are associated with elevated serum proinflammatory cytokines among pregnant women. Brain Behav. Immun..

[8] Osborne L.M., Monk C. (2013). Perinatal depression—The fourth inflammatory morbidity of pregnancy?: Theory and literature review. Psychoneuroendocrinology.

[9] Ravi M., Bernabe B., Michopoulos V. (2022). Stress-related mental health disorders and inflammation in pregnancy: The current landscape and the need for further investigation. Front. Psychiatry.

[10] Baskin R., Hill B., Jacka F.N., O’Neil A., Skouteris H. (2015). The association between diet quality and mental health during the perinatal period. A systematic review. Appetite.

[11] Singh A., Trumpff C., Genkinger J., Davis A., Spann M., Werner E., Monk C. (2017). Micronutrient dietary intake in Latina pregnant adolescents and its association with level of depression, stress, and social support. Nutrients.

[12] Hornig D. (1975). Distribution of ascorbic acid, metabolites and analogues in man and animals. Ann. N. Y. Acad. Sci..

[13] Englard S., Seifter S. (1986). The biochemical functions of ascorbic acid. Annu. Rev. Nutr..

[14] May J.M. (2012). Vitamin C transport and its role in the central nervous system. Subcell. Biochem..

[15] Scaife A.R., McNeill G., Campbell D.M., Martindale S., Devereux G., Seaton A. (2006). Maternal intake of antioxidant vitamins in pregnancy in relation to maternal and fetal plasma levels at delivery. Br. J. Nutr..

[16] Morse E.H., Clarke R.P., Keyser D.E., Merrow S.B., Bee D.E. (1975). Comparison of the nutritional status of pregnant adolescents with adult pregnant women. I. Biochemical findings. Am. J. Clin. Nutr..

[17] Levine M., Conry-Cantilena C., Wang Y., Welch R.W., Washko P.W., Dhariwal K.R., Park J.B., Lazarev A., Graumlich J.F., King J. (1996). Vitamin C pharmacokinetics in healthy volunteers: Evidence for a recommended dietary allowance. Proc. Natl. Acad. Sci. USA.

[18] Kinsman R.A., Hood J. (1971). Some behavioral effects of ascorbic acid deficiency. Am. J. Clin. Nutr..

[19] Moritz B., Schmitz A.E., Rodrigues A.L.S., Dafre A.L., Cunha M.P. (2020). The role of vitamin C in stress-related disorders. J. Nutr. Biochem..

[20] Yosaee S., Keshtkaran Z., Abdollahi S., Shidfar F., Sarris J., Soltani S. (2021). The effect of vitamin C supplementation on mood status in adults: A systematic review and meta-analysis of randomized controlled clinical trials. Gen. Hosp. Psychiatry.

[21] Jafarnejad S., Boccardi V., Hosseini B., Taghizadeh M., Hamedifard Z. (2018). A meta-analysis of randomized control trials: The impact of vitamin C supplementation on serum CRP and serum hs-CRP concentrations. Curr. Pharm. Des..

[22] Ellulu M.S., Rahmat A., Patimah I., Khaza’ai H., Abed Y. (2015). Effect of vitamin C on inflammation and metabolic markers in hypertensive and/or diabetic obese adults: A randomized controlled trial. Drug. Des. Devel. Ther..

[23] Leonard B., Maes M. (2012). Mechanistic explanations how cell-mediated immune activation, inflammation and oxidative and nitrosative stress pathways and their sequels and concomitants play a role in the pathophysiology of unipolar depression. Neurosci. Biobehav. Rev..

[24] Bradley H.A., Campbell S.A., Mulder R.T., Henderson J.M.T., Dixon L., Boden J.M., Rucklidge J.J. (2020). Can broad-spectrum multinutrients treat symptoms of antenatal depression and anxiety and improve infant development? Study protocol of a double blind, randomized, controlled trial (the ‘NUTRIMUM’ trial). BMC Pregnancy Childbirth.

[25] Popper C.W. (2014). Single-micronutrient and broad-spectrum micronutrient approaches for treating mood disorders in youth and adults. Child. Adolesc. Psychiatr. Clin. N. Am..

[26] Bailey R.L., Miller P.E., Mitchell D.C., Hartman T.J., Lawrence F.R., Sempos C.T., Smiciklas-Wright H. (2009). Dietary screening tool identifies nutritional risk in older adults. Am. J. Clin. Nutr..

[27] Cox J.L., Holden J.M., Sagovsky R. (1987). Detection of postnatal depression. Development of the 10-item Edinburgh Postnatal Depression Scale. Br. J. Psychiatry.

[28] Berk M., Ng F., Dodd S., Callaly T., Campbell S., Bernardo M., Trauer T. (2008). The validity of the CGI severity and improvement scales as measures of clinical effectiveness suitable for routine clinical use. J. Eval. Clin. Pract..

[29] Williams J.B., Kobak K.A. (2008). Development and reliability of a structured interview guide for the Montgomery Asberg Depression Rating Scale (SIGMA). Br. J. Psychiatry.

[30] Lovibond P.F., Lovibond S.H. (1995). The structure of negative emotional states: Comparison of the Depression Anxiety Stress Scales (DASS) with the Beck Depression and Anxiety Inventories. Behav. Res. Ther..

[31] Lovibond S.H., Lovibond P.F. (1995). Manual for the Depression Anxiety Stress Scales.

[32] Spitzer R.L., Kroenke K., Williams J.B., Löwe B. (2006). A brief measure for assessing generalized anxiety disorder: The GAD-7. Arch. Intern. Med..

[33] Pullar J.M., Bayer S., Carr A.C. (2018). Appropriate handling, processing and analysis of blood samples is essential to avoid oxidation of vitamin C to dehydroascorbic acid. Antioxidants.

[34] Carr A.C., Pullar J.M., Moran S., Vissers M.C. (2012). Bioavailability of vitamin C from kiwifruit in non-smoking males: Determination of ‘healthy’ and ‘optimal’ intakes. J. Nutr. Sci..

[35] Zhou X., Fragala M.S., McElhaney J.E., Kuchel G.A. (2010). Conceptual and methodological issues relevant to cytokine and inflammatory marker measurements in clinical research. Curr. Opin. Clin. Nutr. Metab. Care.

[36] Rowe S., Carr A.C. (2020). Global vitamin C status and prevalence of deficiency: A cause for concern?. Nutrients.

[37] Carr A.C., Rowe S. (2020). Factors affecting vitamin C status and prevalence of deficiency: A global health perspective. Nutrients.

[38] Carr A.C., Block G., Lykkesfeldt J. (2022). Estimation of vitamin C intake requirements based on body weight: Implications for obesity. Nutrients.

[39] Moya-Alvarez V., Koyembi J.J., Kayé L.M., Mbecko J.R., Sanke-Waîgana H., Djorie S.G., Nyasenu Y.T., Mad-Bondo D., Kongoma J.B., Nakib S. (2021). Vitamin C levels in a Central-African mother-infant cohort: Does hypovitaminosis C increase the risk of enteric infections?. Matern. Child. Nutr..

[40] Coker S.J., Smith-Díaz C.C., Dyson R.M., Vissers M.C.M., Berry M.J. (2022). The epigenetic role of vitamin C in neurodevelopment. Int. J. Mol. Sci..

[41] Rumbold A., Ota E., Nagata C., Shahrook S., Crowther C.A. (2015). Vitamin C supplementation in pregnancy. Cochrane Database Syst. Rev..

[42] Zhang C., Williams M.A., King I.B., Dashow E.E., Sorensen T.K., Frederick I.O., Thompson M.L., Luthy D.A. (2002). Vitamin C and the risk of preeclampsia—Results from dietary questionnaire and plasma assay. Epidemiology.

[43] Carr A.C., Lykkesfeldt J. (2021). Discrepancies in global vitamin C recommendations: A review of RDA criteria and underlying health perspectives. Crit. Rev. Food Sci. Nutr..

[44] Smith Fawzi M.C., Kaaya S.F., Mbwambo J., Msamanga G.I., Antelman G., Wei R., Hunter D.J., Fawzi W.W. (2007). Multivitamin supplementation in HIV-positive pregnant women: Impact on depression and quality of life in a resource-poor setting. HIV Med..

[45] Oliveira K.F., Cunha D.F., Weffort V.R. (2011). Analysis of serum and supplemented vitamin C and oxidative stress in HIV-infected children and adolescents. J. Pediatr. (Rio J.).

[46] Rucklidge J.J., Bradley H.A., Campbell S., Heaton J.L., Mulder R.T., Henderson J., Dixon L., Moltchanova E. (2022). Vitamins and minerals treat antenatal depression and improve birth and infant development: Results of the double-blind NutriMum RCT [Abstract]. J. Am. Acad. Child. Adolesc. Psychiatry.

[47] Bornstein S.R., Yoshida-Hiroi M., Sotiriou S., Levine M., Hartwig H.G., Nussbaum R.L., Eisenhofer G. (2003). Impaired adrenal catecholamine system function in mice with deficiency of the ascorbic acid transporter (SVCT2). FASEB J..

[48] Padayatty S.J., Doppman J.L., Chang R., Wang Y., Gill J., Papanicolaou D.A., Levine M. (2007). Human adrenal glands secrete vitamin C in response to adrenocorticotrophic hormone. Am. J. Clin. Nutr..

[49] Hooper M.H., Carr A., Marik P.E. (2019). The adrenal-vitamin C axis: From fish to guinea pigs and primates. Crit. Care.

[50] Marik P.E. (2020). Vitamin C: An essential “stress hormone” during sepsis. J. Thorac. Dis..

[51] Simon L., Gauvin F., Amre D.K., Saint-Louis P., Lacroix J. (2004). Serum procalcitonin and C-reactive protein levels as markers of bacterial infection: A systematic review and meta-analysis. Clin. Infect. Dis..

